# A Silent Vasculitis With a Loud Presentation: Takayasu Arteritis Causing Intracerebral Hemorrhage

**DOI:** 10.7759/cureus.105244

**Published:** 2026-03-14

**Authors:** Sriram R, Sukrit Nanda M, Sahasyaa Adalarasan, Yogesh Subramanian, Karthigeyan TS, Jayaprakash N, Hariharan C

**Affiliations:** 1 Internal Medicine, Madras Medical College, Chennai, IND

**Keywords:** aneurysm, ich, rare, takayasu, young hypertensive

## Abstract

Takayasu arteritis is a chronic large-vessel vasculitis affecting the aorta and its major branches, most commonly in young women. Neurological involvement is well recognized but typically manifests as ischemic stroke due to arterial stenosis or occlusion. Intracerebral hemorrhage as the initial presentation of Takayasu arteritis is rare and is usually related to severe secondary hypertension or extensive vascular disease. We describe a 27-year-old woman who presented with acute left-sided hemiplegia and facial asymmetry and was found to have a right gangliocapsular intracerebral hemorrhage. She had a background of poorly controlled hypertension and chronic kidney disease without a prior diagnosis of vasculitis. Clinical examination revealed multiple peripheral vascular bruits, prompting further evaluation. CT pan-aortography demonstrated multiple saccular aneurysms involving the infrarenal abdominal aorta and bilateral common iliac arteries, consistent with Numano type IV Takayasu arteritis. The patient underwent surgical intervention followed by immunosuppressive therapy, with stable neurological and clinical status at the three-month follow-up. This highlights the importance of a careful bedside examination during patient examination in order to potentially save a patient's life from an underlying illness.

## Introduction

Takayasu arteritis is a granulomatous large-vessel vasculitis that predominantly affects women under the age of 50, having a predilection for the aorta and its major branches [1]. The disease is characterized by progressive arterial wall inflammation, due to antibodies against the vessel wall components, leading to stenosis, occlusion, aneurysm formation, or secondary end-organ ischemia [2]. Owing to its insidious onset and nonspecific early symptoms, diagnosis is often delayed until advanced vascular involvement or catastrophic complications occur.

The common clinical manifestations of Takayasu arteritis include constitutional symptoms in the early inflammatory phase, followed by vascular features such as upper limb claudication, diminished or absent peripheral pulses, blood pressure discrepancies, arterial bruits, and features of renovascular hypertension [3]. Despite these hallmark signs, the diagnosis may be overlooked in the absence of a high index of suspicion or proper examination.

Stroke is a recognized neurological complication of Takayasu arteritis [4]; however, it most commonly results from arterial stenosis, occlusion, or thromboembolism involving carotid or vertebrobasilar circulation. In contrast, intracerebral hemorrhage is a relatively uncommon cause of stroke in young adults and is typically associated with uncontrolled hypertension, vascular malformations, or coagulopathies [5]. When intracerebral hemorrhage occurs in patients with Takayasu arteritis, it is usually secondary to severe renovascular hypertension or rupture of associated aneurysms, making it a pretty rare but clinically significant presentation.

We report a case of a young woman who presented to the Emergency OPD of the Rajiv Gandhi Government General Hospital with intracerebral hemorrhage as the initial manifestation of Takayasu arteritis, in whom careful clinical examination revealed multiple vascular bruits that prompted further evaluation. This rare case highlights the value of a careful and proper vascular examination in uncovering a silent vasculitis, potentially saving their life.

## Case presentation

A 27-year-old woman presented to the emergency department with an acute onset of weakness of the left upper and lower limbs, accompanied by deviation of the mouth to the right. There was no associated headache, loss of consciousness, seizures, trauma, visual disturbances, or vomiting. There was no past or family history suggestive of connective tissue disease or vasculitis. She was a known case of systemic hypertension for the past eight years and chronic kidney disease, with a history of intermittent hemodialysis, the last session having been performed four months prior to presentation. However, no prior medical records were available, and the patient was admitted due to poor compliance with anti-hypertensive therapy and follow-up. No other significant chronic diseases were reported by the patient.

On examination, the patient was pale, with a recorded blood pressure of 190/100 mmHg. Neurological examination revealed hypotonia of the left upper and lower limbs, with power graded as 0/5 in both. A right-sided upper-motor neuron facial palsy was noted. Higher mental functions were intact, and there were no signs of meningeal irritation. Peripheral vascular examination was remarkable for a palpable thrill over the left brachiocephalic region, a bruit over the left subclavian area, and bilateral renal artery bruits. Prominent abdominal aortic pulsations were noted. The remainder of the general physical and systemic examination was unremarkable.

**Figure 1:**
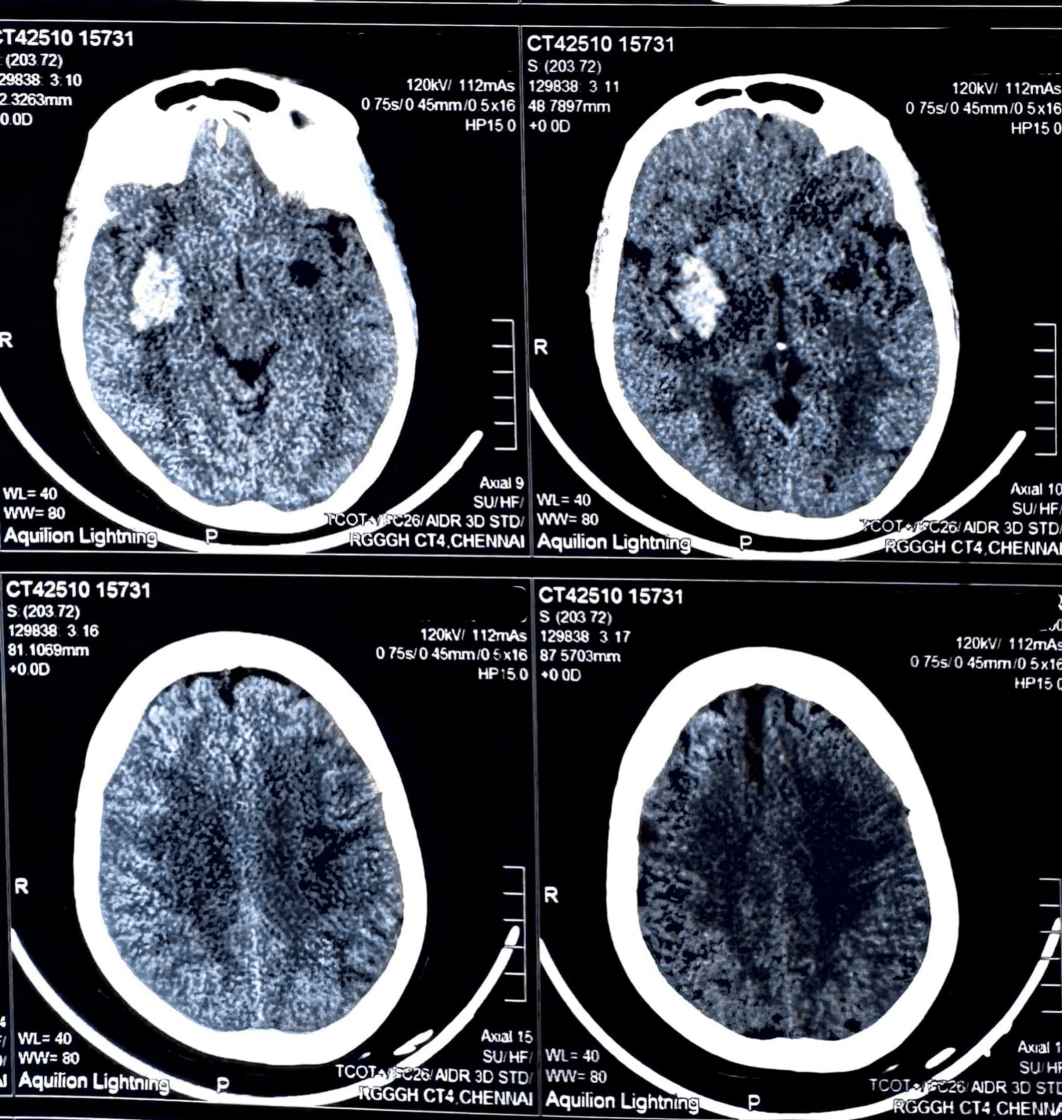
CT brain of the patient at the time of admission

Non-contrast computed tomography (CT) of the brain revealed an acute intracerebral hemorrhage measuring approximately 3.8 × 2.4 × 3.1 cm, located in the right gangliocapsular region with extension into the anterior temporal lobe (Figure 1). Surrounding perilesional edema with effacement of adjacent sulcal spaces and compression of the right lateral ventricle was noted, with a midline shift of approximately 2 mm toward the left. In addition, a chronic infarct was seen involving the left corona radiata and lentiform nucleus.

**Figure 2:**
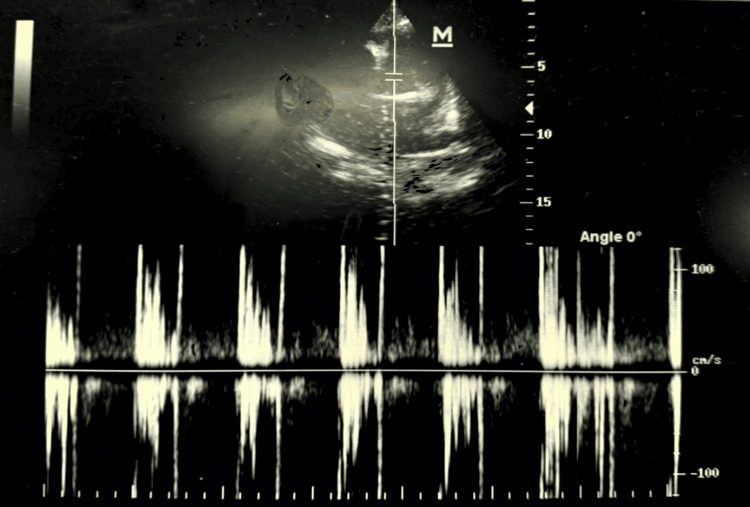
ECHO finding the patient showing a mild mitral regurgitation ECHO: echocardiogram

Laboratory investigations revealed microcytic hypochromic anemia with a hemoglobin level of 8.2 g/dL and a mean corpuscular volume of 76 fL. erythrocyte sedimentation rate (ESR) stood at 62 mm/hr. The summary of the hemogram is depicted below (Table 1). Liver function tests, renal function tests, and capillary blood glucose levels were within normal limits at admission. Transthoracic echocardiography revealed mild mitral regurgitation with preserved ventricular function (Figure 2). Abdominal ultrasonography demonstrated bilateral grade 3 renal parenchymal disease. Doppler evaluation showed monophasic flow in both arteries with an elevated renal resistive index (>0.70), suggestive of significant renovascular involvement.

**Table 1:** Hemogram of the patient Hb: Hemoglobin, PCV: Packed cell volume, MCHC: Mean corpuscular hemoglobin concentration, MCV: Mean cell volume, ESR: Erythrocyte sedimentation rate, CRP: C-reactive protein

Parameter	Value (in units)	Reference range
Hb	8.2 g/dL	11-15.5
PCV	27.40%	36-48
MCHC	23.8 g/dL	32-36
MCV	76 fL	80-100
ESR	62 mm/hr	0-20
CRP	42.9 mg/L	0-5

**Figure 3:**
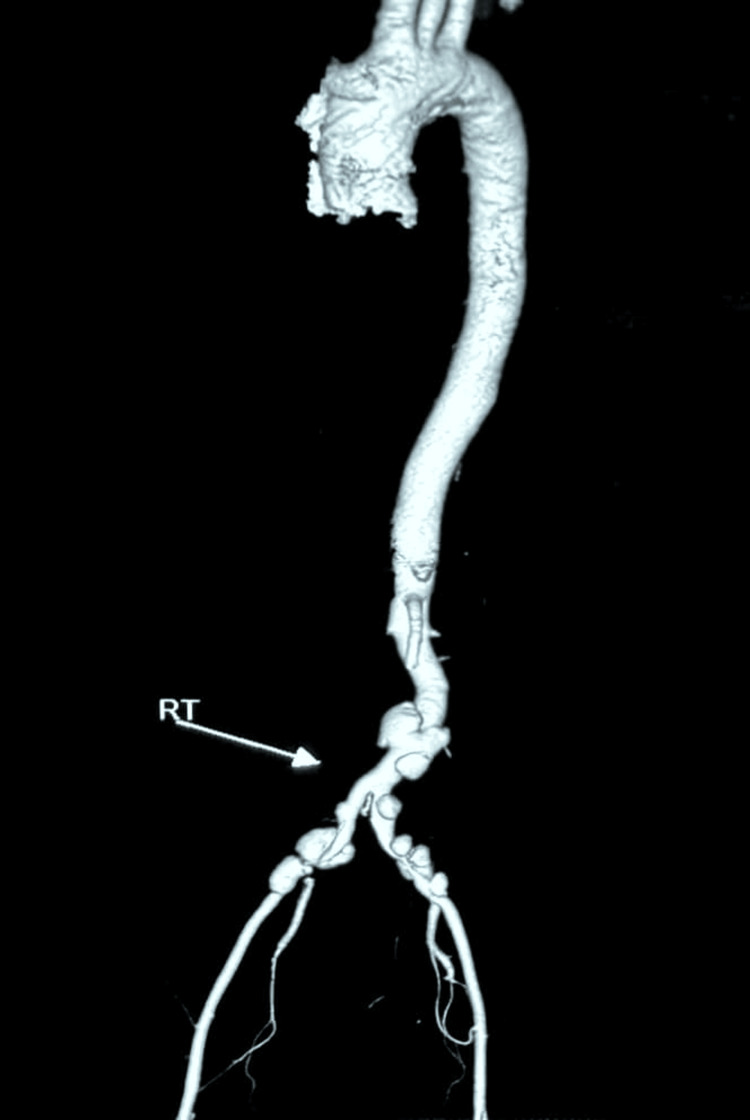
Aortogram of the patient taken during the admission workup showing several abdominal aortic aneurysms

The standard management for hemorrhagic stroke (according to the availability of drugs and the routine of the hospital) was initiated prior to these investigations, which included nifedipine 10 mg three times a day (TDS), prazosin 2.5 mg twice daily (BD), metoprolol 25 mg BD, calcium 300 mg TDS, and atorvastatin 10 mg at bedtime (HS). In view of the patient’s young age, refractory hypertension, chronic kidney disease, and presence of multiple vascular bruits, a vasculitic etiology was suspected, and further evaluation was undertaken. CT pan-aortography revealed multiple saccular aneurysms with mural thrombi involving the infrarenal abdominal aorta and bilateral common iliac arteries (Figure 3). These findings were consistent with Takayasu arteritis, classified as Type IV disease according to the Numano classification [6].

The patient was subsequently evaluated by the vascular surgery team and underwent surgical intervention (abdominal endovascular aneurysmal repair) for aneurysmal disease. Postoperatively, she was initiated on disease-specific therapy for Takayasu arteritis, including systemic corticosteroids (dexamethasone). Her hospital course was uneventful, and she showed gradual neurological stabilization. At the three-month follow-up, the patient remained clinically stable with no further hospital admissions or new neurological complaints.

## Discussion

Takayasu arteritis is a chronic large-vessel vasculitis that predominantly affects young women and typically presents with constitutional symptoms, limb claudication, or features of arterial insufficiency [1]. Intracranial involvement is not unheard of in the case of Takayasu arteritis, considering about 44% of the patients report CNS symptoms [7]. Neurological involvement, when present, is most often ischemic in nature and results from stenotic or occlusive disease of supra-aortic vessels.

Intracerebral hemorrhage (ICH) as an initial manifestation of Takayasu arteritis is distinctly uncommon and is usually secondary to long-standing, poorly controlled renovascular hypertension or rupture of associated aneurysms [5]. In the literature, there seem to be only four prior reported cases of non-aneurysmal ICH manifesting as the primary complaint in aortitis syndromes or Takayasu arteritis.

A recent case report includes a 15-year-old female with similar complaints and loss of appetite presenting with a subarachnoid hemorrhage and ICH [8]. A pediatric female with both ICH and abdominal aorta involvement has also been reported [9]. Two other young women, aged 21 and 32 years, presented similarly to the present case [10,11]. A 48-year-old female with a long-standing disease also presented with a subarachnoid hemorrhage (SAH) (from aneurysms) and abdominal involvement [12]. Table 2 summarizes all the cases of non-aneurysmal ICH in Takayasu arteritis.

**Table 2:** Tabular comparison of the literature specific to the present case

Characteristics	Case 1	Case 2	Case 3	Case 4	Case 5
Age	15	11	21	32	27
Duration of disease	2 months	Newly diagnosed	10 years	10 years	Newly diagnosed
Chief complaint(s)	Weakness, numbness and loss of appetite	Hemiparesis, facial asymmetry	Headache, abdominal pain	Weakness, numbness, vomiting	Hemiparesis, facial asymmetry
Involvement of abdominal aorta	No	Yes	Yes	No	Yes
Location of bleed	Subarachnoid	Intraparenchymal	Intraparenchymal	Intraparenchymal	Intraparenchymal
Management	Evacuation of hematoma, medical treatment	Medical treatment	Medical treatment	Medical treatment	Medical treatment, EVAR
1 month follow-up	Patient under control	Patient under control	Patient under control	Patient under control	Patient under control
Reference	[8]	[9]	[10]	[11]	Present case

The limitations of the present case include the lack of detailed immunologic or genetic investigations. This report underscores the importance of considering secondary causes of hypertension and stroke in young patients and highlights the diagnostic value of meticulous peripheral vascular examination in uncovering an otherwise silent systemic vasculitis. Future improvements in screening programs and education about rare differentials, such as the current case report, can aid in the improvement of patient care.

## Conclusions

Takayasu arteritis is an uncommon but important cause of secondary hypertension and cerebrovascular disease in young women. Although ischemic stroke is the more frequently encountered neurological manifestation, intracerebral hemorrhage may rarely occur as the initial presentation, most often as a consequence of uncontrolled renovascular hypertension or extensive vascular involvement. This case adds to the limited literature describing non-aneurysmal intracerebral hemorrhage as the first manifestation of Takayasu arteritis.

The present report underscores the critical role of a thorough bedside vascular examination in young patients presenting with stroke, particularly in the presence of refractory hypertension or renal dysfunction. Early suspicion and timely imaging can facilitate prompt diagnosis and definitive management, potentially preventing further catastrophic vascular events. Increased awareness of such atypical presentations among general physicians may lead to earlier recognition of this otherwise silent vasculitis and improved patient outcomes. An earlier recognition could also prevent severe complications or even death of the patient.
